# Potential of AON therapy targeting AASS: A promising molecular therapy for pyridoxine-dependent epilepsy

**DOI:** 10.1016/j.omtn.2025.102767

**Published:** 2025-11-18

**Authors:** Mustafa A. Salih

**Affiliations:** 1 Professor and Consultant Pediatric Neurologist, Health Sector, King Abdulaziz City for Science and Technology, Riyadh, Saudi Arabia

## Main text

Pyridoxine-dependent epilepsy (PDE) is a rare autosomal recessive disorder, associated with pathogenic variants in *ALDH7A1* leading to aberrant lysine degradation and accumulation of neurotoxic metabolites. The condition is characterized by epileptic seizures, typically occurring soon after birth, which are unresponsive to standard antiseizure medications (ASMs) and respond only to pyridoxine.^1^^,^^2^ Seizures may respond initially to standard ASM in some patients.^3^ Despite initiation of early treatment and seizure control, global developmental delay and/or intellectual disability have been reported in 75% of PDE patients.^2^^,^^4^^,^^5^ Current treatments, including lysine-restricted diets aiming to reducing toxic metabolite accumulation by lysine reduction and supplementation of arginine, a competitive inhibitor for lysine to cross the blood-brain barrier, only partially alleviate intellectual disability.^6^ In the study highlighted in this commentary, Schuurmans and colleagues^6^ explored underlying mechanisms and potential novel therapies for PDE.

The neurometabolic dysfunction of PDE results from ALDH7A1 enzyme deficiency, caused by pathogenic variants in *ALDH7A1*, which converts 2-aminoadipic-6-semialdehyde (α-AASA) to 2-aminoadipic acid (AAA) in the lysine degradation pathway. The resulting enzyme deficiency leads to neurotoxic metabolite accumulation including α-AASA, 1-piperideine-6-carboxylic acid (P6C), and pipecolic acid. Excessive P6C combines with pyridoxal phosphate (PLP) resulting in lowered availability. Inactivation of PLP, which is the active form of vitamin B6 (pyridoxine) and constitutes an important cofactor for enzymes involved in GABA synthesis, is thought to be the underlying cause of the pyridoxine-responsive seizures. The current available treatment strategies for PDE include supplementation of vitamin B6 (pyridoxine) to control seizures and dietary interventions aimed at reducing the toxic metabolite accumulation through lysine reduction and arginine supplementation.^7^ However, since lysine is an essential amino acid that cannot be completely removed, these strategies proved to have limited efficacy.

Schuurmans and colleagues^6^ explored an alternative therapy by downregulating alpha-aminoadipic semialdehyde synthase (AASS), the first enzyme of the lysine catabolism, which harbors the lysine-ketoglutarate reductase (LKR) and the saccharopine dehydrogenase domains that convert lysine to alpha-aminoadipic semialdehyde synthase (AASS). Complete deficiency of AASS, particularly when the LKR domain is affected, manifests as hyperlysinemia type I, a disease where lysine accumulation is associated with none or mild clinically relevant phenotypes.^8^ Antisense oligonucleotides (AONs), which modulate degradation of mRNA transcripts, were used for partial inhibition of AASS. Patient-derived human induced pluripotent stem cells (hiPSCs) were used as a strategy allowing the study of cell-type-specific pathophysiological mechanisms underlying PDE and for testing the new genetic therapeutic strategy. They were subsequently differentiated into astrocytes, which constitute the key regulators of metabolic homeostasis and are the primary source of ALDH7A1 in the brain. Analysis of patient-derived hiPSC revealed gene expression changes consistent with increased oxidative stress, elevated PDE biomarkers, increased reactive oxygen species levels and lipid peroxidation, and suggested mitochondrial dysfunction evidenced by dysregulated oxygen consumption rates. Downregulating AASS using AONs resulted in alleviation of these pathological phenotypes and highlighted a promising molecular therapy for PDE as well as other inherited metabolic disorders.

## Declaration of interests

The author declares no competing interests.
